# Abdominal obesity is associated with heart disease in dogs

**DOI:** 10.1186/1746-6148-10-131

**Published:** 2014-06-13

**Authors:** Naris Thengchaisri, Wutthiwong Theerapun, Santi Kaewmokul, Amornrate Sastravaha

**Affiliations:** 1 Department of Companion Animal Clinical Sciences, Faculty of Veterinary Medicine, Kasetsart University, 50 Pahonyothin Rd., Lat Yao, Chatuchak, Bangkok 10900, Thailand; 2 Department of Physiology, Faculty of Veterinary Medicine, Kasetsart University, Bangkok 10900, Thailand

**Keywords:** Dogs, Abdominal obesity, Heart disease, Receiver operating characteristic, Waist circumference, Computed tomography

## Abstract

**Background:**

The relationship between overall obesity and fat distribution in dogs and the development of heart disease is unclear. In the present study we evaluated the association between overall obesity and fat distribution and clinical heart disease by morphometric and computed tomography (CT)-based measurements. Body condition score (BCS), modified body mass index (MBMI, kg/m^2^), waist-to-hock-to-stifle distance ratio (WHSDR), waist-to-ilium wing distance ratio (WIWDR), and waist-to-truncal length ratio (WTLR) were compared between dogs with (n = 44) and without (n = 43) heart disease using receiver operating characteristic (ROC) analysis. Intra-abdominal fat (IAF) and subcutaneous fat (SQF) were measured in dogs with (n = 8) and without (n = 9) heart disease at the center of the fourth and fifth lumbar vertebrae by CT.

**Results:**

BCS was similar between heart disease and healthy groups (3.6 ± 0.2 vs. 3.3 ± 0.1, P = 0.126). The following morphometric measurements were greater in the heart disease group compared with healthy canines: MBMI (65.0 ± 4.5 vs. 52.5 ± 3.7 kg/m^2^, respectively, P = 0.035); WIWDR (4.1 ± 0.1 vs. 3.1 ± 0.1, P < 0.01); and WTLR (1.25 ± 0.04 vs. 1.05 ± 0.04, P < 0.01). However, there was no significant difference in WHSDR (3.6 ± 0.1 vs. 3.7 ± 0.2, P = 0.875). Interestingly, IAF was significantly increased in dogs with heart disease compared with healthy dogs (23.5 ± 1.5% vs. 19.4 ± 1.2%, P = 0.039) whereas SQF was similar between two groups (35.5 ± 2.7% vs. 38.6 ± 3.5%, P = 0.496). Of the five morphometric indices studied, WIWDR and WTLR provided acceptable discrimination for diagnosing heart disease in dogs, with areas under the ROC curve of 0.778 (95% confidence interval [CI]:0.683-0.874) and 0.727 (95% CI:0.619-0.835), respectively.

**Conclusions:**

Our data indicate that abdominal obesity, rather than overall obesity, is associated with heart disease in dogs. Measurements of both WIWDR and WTLR are particular useful for detection of an abdominal obesity in dogs.

## Background

Clinical assessments of human patients have emphasized the importance of accumulated visceral fat rather than peripheral fat as one of the underlying causes of heart disease [1,2]. Visceral obesity is also associated with insulin insensitivity [3], which can result in various metabolic abnormalities and health complications. Dogs commonly become overweight as a result of unintentional overfeeding and approximately 24-30% of the pet dog population is estimated to be overweight [4]. Various diseases in dogs, including heart disease [5,6], metabolic dysregulation (i.e., diabetes) [7,8], osteoarthritis [9], urinary tract and reproductive disorders [10,11], and neoplasia [12] are linked to being overweight.

Measurement of obesity including relative body weight (BW) [8], body condition score (BCS) [13-15], morphometric analysis [15], and dimensional evaluation (performed by tape measurement) [15], provides a convenient evaluation of a dog’s general body composition [14,15]. However, visceral fat cannot be assessed with these measurements. In clinical settings, CT provides a minimally invasive method for peripheral and visceral fat measurement [16,17]. In humans, altered body conformation, especially the presence of abdominal obesity, might enhance the risk of heart disease [18]. In addition, heart disease is associated with numerous morphometric and dimensional measurements. Clinical appraisal of canine abdominal obesity allows veterinarians to warn owners to pay more attention to their pet’s obesity problem and the resulting increased risk of developing heart disease. The present study comprehensively compared BCS, body shape, and visceral fat between healthy dogs and those with heart disease.

## Methods

### Animal subjects

We evaluated 87 dogs that were patients of Kasetsart University Veterinary Teaching Hospital over a 2-year period (October 2005 to October 2007). The protocol was reviewed and approved by Kasetsart University Animal Care and Use Committee, and informed consent was obtained from all owners. Of these 87 dogs, 43 were physically healthy and 44 were determined to have heart disease and visited the heart clinic for physical check-ups (Table 1). In the healthy group, there were 11 (25.6%) Poodle, 7 (16.3%) mixed-breed dogs, 7 (16.3%) Shih Tzu, 3 (7%) Golden Retriever, 2 (4.7%) Chihuahua, 2 (4.7%) English Cocker, 2 (4.7%) Pugs, and 9 (20.9%) other breeds. In the heart disease group, there were 18 (40.9%) mixed-breed dogs, 9 (20.5%) Poodles, 2 (4.5%) Cocker Spaniel, 2 (4.5%) Miniature Pincher, 2 (4.5%) Pomeranian, 2 (4.5%) Shih Tzu, and 9 (20.5%) other breeds. Sex distribution (dogs with heart disease: 19 sexually intact males, 7 castrated males, 10 sexually intact females, and 8 spayed females; control dogs: 19 sexually intact males, 4 castrated males, 17 sexually intact females, and 3 spayed females) did not differ significantly between groups. Of the 44 dogs with heart disease, 17 had cardiac enlargement, 13 had valvular heart disease, 7 had cardiac arrhythmia, 6 had congestive heart failure, and 1 had cardiomyopathy. In addition, among heart disease dogs, four were positive for heartworm.

**Table 1:** General characteristics of the subject dogs

**Classification**	**Healthy**	**Heart disease**
N	43	44
Age		
1-5 years	36	7
>5 years	7	37
Gender		
Male	23	26
Female	20	18
Body size		
Small (<12 kg)	36	28
Medium (12–24 kg)	4	12
Large (>24 kg)	3	4
BCS	3.3 ± 0.1	3.6 ± 0.2
MBMI (BW (kg)/[TL (m)]^2^)	52.5 ± 3.7	65.0 ± 4.5*
WHSDR	3.7 ± 0.2	3.6 ± 0.1
WIWDR	3.1 ± 0.1	4.1 ± 0.1**
WTLR	1.05 ± 0.04	1.25 ± 0.04**

*p < 0.05, **p < 0.01. *BCS,* body condition score; *BW*, body weight; *MBMI,* modified body mass index; *BW,* body weight; *TL,* truncal length; *WHSDR,* waist-to-hock-to-stifle distance ratio; *WIWDR,* waist-to-ilium wing distance ratio; *WTLR,* waist-to-truncal length ratio.

### Body condition score and morphometric measurement

Various clinical assessments of canine body composition were performed. Body condition score was recorded using a five-point scale (1 = very thin, 2 = underweight, 3 = ideal weight, 4 = overweight, and 5 = obese) [14,15]. Evaluation of body dimensions was performed by tape measurement. Truncal length (TL, cm) the length of the dog body measured from the front of the chest at the shoulder level to the point of buttock. The body mass index (BMI) is a measurement of human body shape based on mass and height (kg/m^2^). Because dogs walk on four legs, truncal length rather than height was used for MBMI calculation. Therefore, the modified body mass index (MBMI) was defined as the dog’s body weight divided by the truncal length in meters squared (BW (kg)/[TL (m)]^2^). Waist circumference (WC, cm) was measured over a dorsal spine of the 4^th^ lumbar vertebrae using a measuring tape wrapping around the dog abdomen approximately midway between the last rib and the iliac crest in a standing position. Ilium wing distance (IWD, cm) is the width between the dorsal iliac crests. Hock-to-stifle distance (HSD, cm) is a distance from the knee joint to the hock joint. Various relative waist circumference measures including waist-to-ilium wing distance ratio (WIWDR), waist-to-truncal length ratio (WTLR) and waist-to-hock-to-stifle distance ratio (WHSDR) were used in the present study.

### Computed tomography

With the owners’ consents, 17 dogs (9 healthy and 8 heart disease dogs) with body condition scores between 4–5 were randomly selected for CT scan for assessment of the subcutaneous and visceral fat. Dogs were fasted for 12 hours prior to the study. They were sedated by intravenous injection of diazepam (0.5 mg/kg) and anesthesia was induced with intravenous propofol (2 mg/kg body weight). After endotracheal intubation, anesthesia was maintained with isoflurane inhalation (2% in 100% oxygen). All dogs were positioned in sternal recumbency before operating a conventional CT scanner (Philips TOMOSCAN CX/Q, the Netherland). The acquisition parameters were: 120 kVP; 200 mA; field of view 240 mm; collimation,10 mm, and scanning time 4 s/rotation. Slices were acquired from the center of the fourth and fifth lumbar vertebrae. To determine the distribution of visceral and subcutaneous fat, digital image data sets obtained from individual dogs were analyzed using a range of −135 to −105 HU, and then intra-abdominal fat (IAF) and subcutaneous fat (SQF) area were measured and normalized to total abdominal area.

### Data analysis

Differences between the two groups were compared using Tukey’s HSD test. Correlations between morphometric measurements in this study were tested with a pair-wise Pearson study. Data were expressed as mean ± S.E and were compared using one-way analysis of variance. Receiver operating characteristic (ROC) analysis was used to compare the performance of BCS, MBMI, WHSDR, WIWDR, and WTLR as indices of central obesity by determining the diagnostic power of the test by measuring the area under the curve (AUC) using STATA12 (Stata Inc., College Station, TX, USA) statistical software. A perfect test is going to have an AUC of 1.0 and an AUC of 0.5 means the test performs no better than chance. Youden’s index (Youden’s J statistic; J = Sensitivity + specificity - 1) was applied to identify the optimal cut-off value of the morphometric measurements that yielded maximum sums from the ROC curves. Logistic regression analysis was used to determine the odds ratios of cardiovascular disease in dogs associated with either WIWDR or WTLR at the optimal cut-off value. All statistical comparisons were two-tailed, and P-values <0.05 were considered statistically significant.

## Results

**Figure 1:**
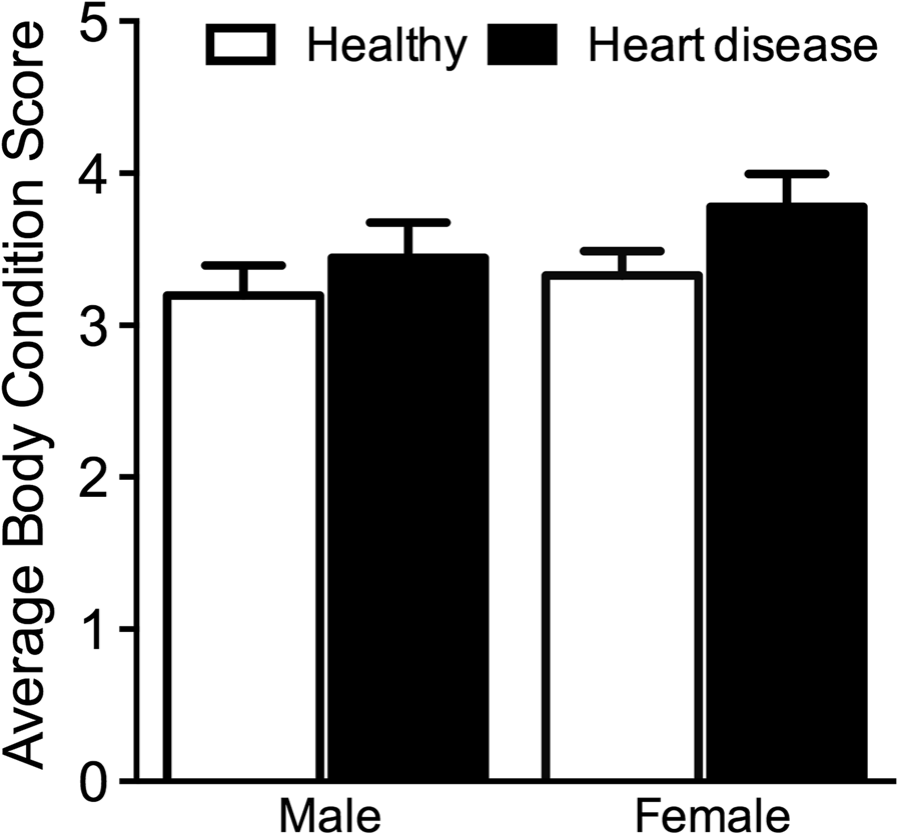
Average body condition score (mean ± SE) of male and female dogs from healthy and heart disease groups.

The average BCS in the heart disease group was slightly higher than that in the healthy group but the difference was not significant (3.6 ± 0.2 vs. 3.3 ± 0.1, P = 0.126). There was also no significant difference in BCS between heart disease and healthy groups when animals were analyzed according to gender: 3.4 ± 0.2 vs. 3.2 ± 0.2 (P = 0.4293) for males and 3.8 ± 0.2 vs. 3.3 ± 0.2 (P = 0.701) for females (Figure 1). The average MBMI in the heart disease group was significantly higher than that in healthy animals (65.0 ± 4.5 vs. 52.5 ± 3.7 kg/m^2^; P = 0.035) (Table 1).

To compare abdominal obesity of healthy versus heart disease dogs, the relative waist circumference was assessed using three different ratios (WIWDR, WHSDR, and WTLR). For WHSDR, no difference was observed between heart disease and healthy groups (3.6 ± 0.1 vs. 3.7 ± 0.2, respectively; P = 0.875). However, dogs with heart disease possessed significantly higher WIWDR (heart disease 4.1 ± 0.1 vs. healthy 3.1 ± 0.1; P < 0.01) and WTLR (heart disease 1.25 ± 0.04 vs. healthy 1.05 ± 0.04; P < 0.01) than healthy dogs (Table 1).

**Figure 2:**
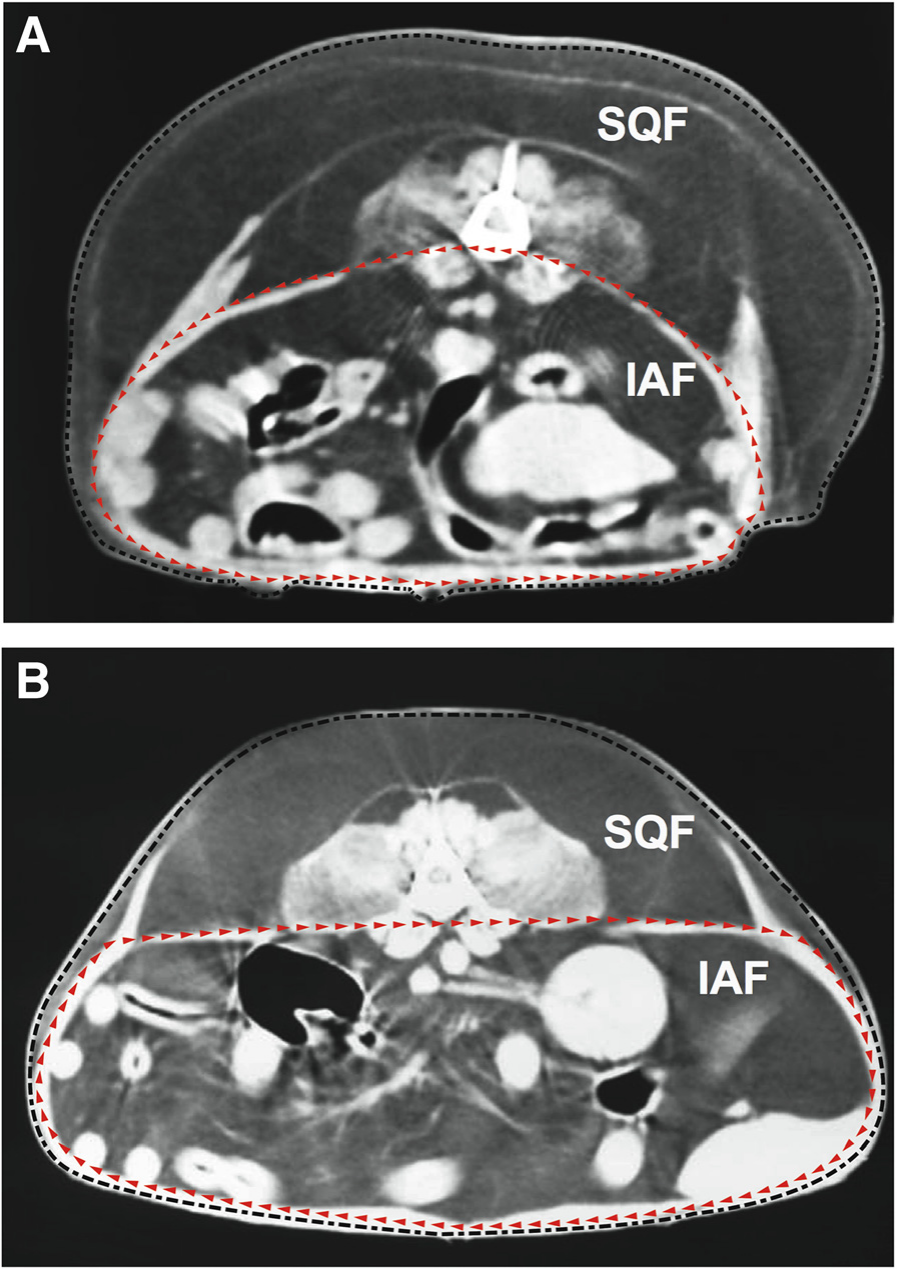
**The cross-sectional area (mean ± SE) of abdominal fat using computed tomography at the fourth and fifth lumbar vertebrae.** The area of intra-abdominal fat (IAF) and subcutaneous fat (SQF) was compared between healthy dogs **(A)** and dogs with heart disease **(B)**. The subcutaneous fat was identified inside the black dashed line and outside the red dashed line. The area of intra-abdominal fat was identified inside the red dashed line.

**Figure 3:**
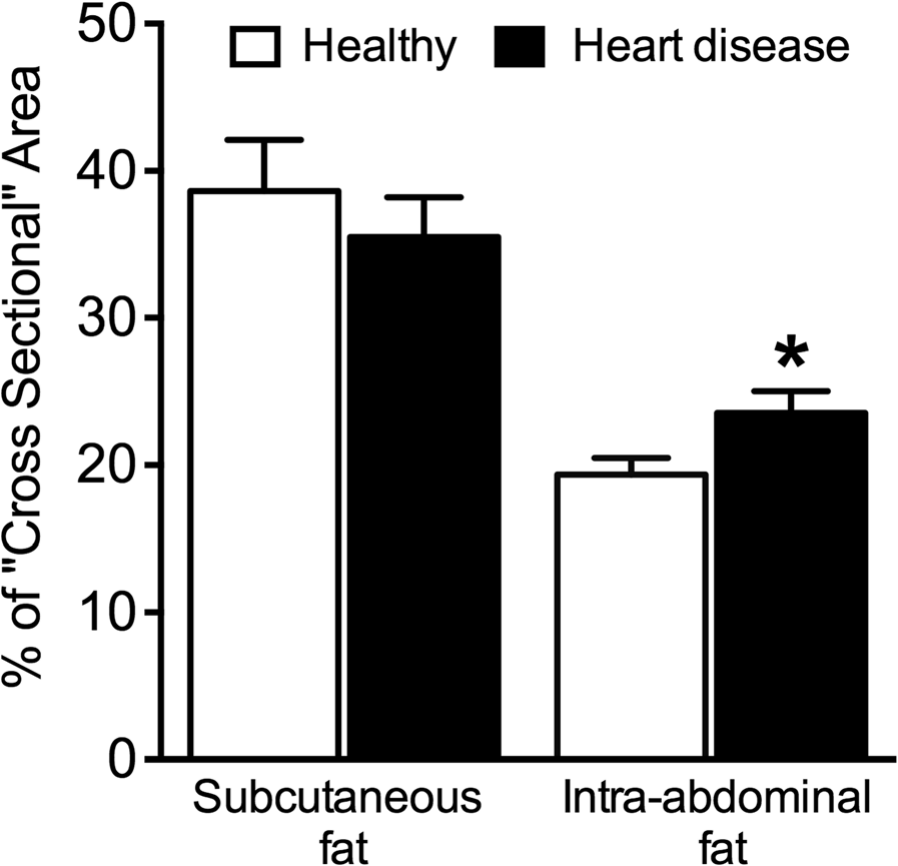
The percentage of cross-sectional areas of subcutaneous and intra-abdominal fat between healthy dogs and dogs with heart disease. *P < 0.05.

Correlation analyses of fat measurements revealed that BCS was positively correlated with MBMI (r = 0.494, P < 0.01), WHSDR (r = 0.553, P < 0.01) and WTLR (r = 0.572, P < 0.01), but not with WIWDR (r = 0.182, P > 0.05) (Table 2). Furthermore, MBMI was significantly correlated with WIWDR (r = 0.303, P < 0.01) and WTLR (r = 0.690, P < 0.01), but not with WHSDR (r = 0.130, P > 0.05) (Table 2).We used CT to assess IAF and SQF at the center of the fourth and fifth vertebrae in dogs with or without heart disease (Figure 2). There was no significant difference in SQF between dogs in the heart disease group and those in the healthy group (35.5 ± 2.7% vs. 38.6 ± 3.5%, respectively; P = 0.50) (Figure 3); however, the amount of IAF of dogs in the heart disease group was significantly higher than that in the healthy group (23.5 ± 1.5% vs. 19.4 ± 1.2%; P = 0.04) (Figure 3). Moreover, the level of SQF was significantly higher than the level of IAF in both the heart disease and the healthy group.

**Table 2:** Correlation coefficient (r) between body condition scores and morphometric measurements

	**MBMI, BW/TL**^**2**^	**WHSDR**	**WIWDR**	**WTLR**
BCS (95% CI)	0.49** (0.32, 0.64)	0.55** (0.39, 0.68)	0.18 (−0.03, 0.38)	0.57** (0.41, 0.70)
MBMI, BW/TL^2^ (95% CI)	-	0.13 (−0.08, 0.33)	0.30** (0.10, 0.48)	0.69** (0.56, 0.79)

**p-value < 0.01, Pearson correlation coefficients. *BCS,* body condition score; *BW*, body weight; *CI,* confidence interval; *MBMI,* modified body mass index; *TL,* truncal length; *WHSDR,* waist-to-hock-to-stifle distance ratio; *WIWDR,* waist-to-ilium wing distance ratio; *WTLR,* waist-to-truncal length ratio.

### Performance of the indices of central obesity

The AUCs from the ROC analyses of BCS, MBMI, WHSDR, WIWDR, and WTLR for predicting the presence of cardiovascular disease in dogs were shown in Table 3. The AUCs from the ROC analyses of MBMI, WIWDR, and WTLR were significant different, compared with the null hypothesis true area of 0.5. The largest area under the ROC curve was obtained using WIWDR (AUC = 0.778; 95% CI:0.683-0.874), followed by WTLR (AUC = 0.727; 95% CI:0.612-0.835) (Table 3).

**Table 3:** The area under the ROC curve of various morphometric indices for cardiovascular risk in dogs

**Morphometric indices**	**AUC (fit model)**	**95% CI**
BCS	0.584	0.466-0.702
MBMI (BW (kg)/[TL (m)]^2^)	0.642*	0.521-0.762
WHSDR	0.477	0.353-0.601
WIWDR	0.778**	0.683-0.874
WTLR	0.727**	0.619-0.835

*p < 0.05, **p < 0.01. *ROC,* receiver operating characteristics; *AUC,* area under the curve; *BCS,* body condition score; *BW*, body weight; *CI*, confidence interval; *MBMI,* modified body mass index; *TL,* truncal length; *WHSDR,* waist-to-hock-to-stifle distance ratio; *WIWDR,* waist-to-ilium wing distance ratio; *WTLR,* waist-to-truncal length ratio.

The cut-off point between sensitivity and specificity curves for WIWDR was 3.6, which also corresponded to the highest Youden’s index (44.82%). The WIWDR cut-off point of 3.6 gave 72.73% sensitivity and 72.09% specificity (Table 4). Dog with WIWDR higher than 3.6 were 7-fold (odds ratios: 6.9 ; 95% CI: 2.7-17.6, p < 0.01) more likely to have cardiovascular diseases than dogs without this factor. The cut-off point between sensitivity and specificity curves for the WTLR was 1.1, which gave 63.64% sensitivity and 67.44% specificity. The optimal cut-off point for WTLR was 1.2, which corresponded to the highest Youden’s index (35.95%). Using a cut-off of 1.2 for the WTLR resulted in a significant improvement in specificity to 81.40%; however, the sensitivity was slightly reduced to 54.55% (Table 4). Dogs with WTLR higher than 1.2 were 5-fold (odds ratios: 5.2; 95% CI: 2.0-13.9, p < 0.01) more likely to have cardiovascular diseases than dogs without this factor.

**Table 4:** Sensitivity and specificity to predict heart disease in canine patients according to different WIWDR and WTLR cut-off points

**CVD risk factors**	**Cut-off point**	**Sensitivity (%)**	**Specificity (%)**	**Positive predictive value (%)**	**Negative predictive value (%)**	**Youden’s index (%)**
WIWDR	3.4	75.00	62.79	67.35	71.05	37.79
	3.5	72.73	69.77	71.11	71.43	42.50
	3.6	72.73	72.09	72.73	72.09	44.82
	3.7	65.91	72.09	70.73	67.39	38.00
	3.8	52.27	76.74	69.70	61.11	29.01
WTLR	0.8	100.00	16.28	55.00	100.00	16.28
	1.0	86.36	51.16	64.41	78.57	37.52
	1.1	63.64	67.44	66.67	64.44	31.08
	1.2	54.55	81.40	75.00	63.64	35.95
	1.4	31.82	88.37	73.68	55.88	20.19

*WIWDR,* waist-to-ilium wing distance ratio; *WTLR,* waist-to-truncal length ratio; *CVD,* cardiovascular disease.

## Discussion

In the present study we compared BCS, MBMI, and abdominal obesity between healthy dogs and dogs affected with heart disease. The average BCS was comparable between healthy dogs and dogs with heart disease; however, the average MBMI was significantly higher in dogs with heart disease than in healthy dogs. Furthermore, the relative waist circumference indices including the WIWDR and the WTLR, but not the WHSDR, were higher in dogs with heart disease compared with healthy dogs, suggesting that dogs with abdominal obesity have a higher chance of developing heart disease. The pair-wise correlation study indicated an association among BCS, MBMI, and morphometric measurements, and suggests a strong association between fat accumulation and body conformation change. Furthermore, the amount of intra-abdominal fat in dogs with heart disease was significantly higher than that in healthy dogs although the amount of subcutaneous fat did not differ significantly between the groups.

The number of dogs with heart disease that were older than 5 years old enrolled in the present study were found more than the healthy group. The different of age distribution between healthy group and heart disease group may be due to the fact that heart diseases are commonly found in the older dogs [19,20]. Due to the fact that dogs may gain weight with aging and desexing that may influence the study results. In the present study, sex distribution did not differ significantly between groups and the majority of the population were intact dogs. Moreover, the body condition scores were comparable between dogs with and without heart diseases groups (Table 1). Measuring the relative abdominal obesity using our study revealed that relative abdominal obesity by measuring WIWDR and WTLR suggested the present of abdominal obesity in dogs with heart disease compared to the healthy group (Table 1).

In humans, central obesity (determined by waist circumference) in association with various morbidities including dyslipidemia [21], hypertension [22], and glucose intolerance [23] is linked to metabolic dysfunction that leads to the development of cardiovascular diseases [24] and diabetes mellitus [25]. Interestingly, canine obesity is also relevant to the development of insulin resistance [7], altered lipid profiles [26], and mild hypertension [7] that could be improved by weight reduction [27]. The results of this CT-based study indicate increased IAF in dogs with heart disease compared with healthy dogs. Despite the evidence of an increase in visceral fat in dogs with heart disease, the pathophysiological roles of visceral fat on increased risk of cardiovascular diseases in dogs remain elusive [28].

Waist circumference has been shown to be an independent risk factor for heart disease in humans [29]. It has been hypothesized that abdominal obesity provides biologic evidence of underlying metabolic disturbances [30]. Therefore, waist circumference has been used in human medicine as a surrogate marker of abdominal fat mass [31] that is associated with cardiometabolic disease risk [21,23]. In veterinary medicine, dogs with abdominal obesity cannot be identified directly from measurement of waist circumference because of variations in body size among different dog breeds. Therefore we used various alternative morphometrics in the present study to assess canine waist circumference including WHSDR, WIWDR, and WTLR. Body condition score was associated with WHSDR and WTLR whereas modified body mass index was associated with WIWDR and WTLR. Thus, WIWDR and WTLR may provide a better assessment of increased relative waist circumference and overall body fat in dogs.

Human studies have identified various anthropometric indices that are cardiovascular disease risk factors including BMI [1,32], waist circumference [1,29,31], waist-to-hip ratio [29], and waist-to-height ratio [32,33]. In the present study, the morphometric indices that provided acceptable discrimination (AUC ≥0.7) between healthy dogs and those with heart disease were WIWDR and WTLR, with AUC for the ROC curve of 0.778 (95% CI:0.683-0.874) and 0.727 (95% CI:0.619-0.835), respectively. Both WIWDR and WTLR provide a simple assessment by which veterinarians can readily identify canine patients at increased risk of developing heart disease. The relative waist circumference may provide a basis for future staging systems for dogs with heart disease, to which further discriminatory variables might be added.

Despite the obvious strength of using WIWDR and WTLR to predict the risk of canine heart disease, it should be noted that a relatively small number of animals were enrolled in this study (87 cases). Waist circumference is widely accepted in human medicine as a strong independent risk factor of heart disease [1,29,31]. Developing novel specific body measurements in dogs for monitoring abdominal obesity will significantly improve the detection rate of dogs at risk of developing cardiovascular diseases. In the present study, measurement of subcutaneous and intra-abdominal fat was performed in only 17 cases, thus limiting the strength of the comparison of relative waist circumference with computed tomographic information. However, the main objective of the present study was to establish the performance of relative waist circumference parameters (WIWDR and WTLR) compared with conventional parameters such as body condition score. Nonetheless, the accumulation of intra-abdominal fat identified by CT was higher in dogs with heart disease compared with healthy dogs, indicating that excess visceral fat is associated with cardiovascular disease in canine patients, similar to humans [21].

### Significant for comparative research

Various hormones, metabolically active proteins and adipokines act as inflammatory mediators, promoting the chronic inflammatory state in obesity [13]. In human, visceral obesity has been associated with noninfectious inflammation [34] that has been linked to development of metabolic syndrome and increase incidence of cardiovascular disease and diabetes [35]. Interestingly, the present study associated the presence of abdominal obesity in dogs with heart disease similar to that found in humans [31,32,36]. The similarities in living environment and the diet of humans and dogs gives rise to the possibility that canine visceral obesity may be a good model for the disease in human patients with abdominal obesity. Nevertheless, the mechanisms of how abdominal obesity leaded to development of heart disease in canine patients was not conducted in the present study. Additional studies of disease pathogenesis, identification of novel non-invasive diagnostic markers, and the development of novel therapeutic agents for management of abdominal obesity for canine patients should be conducted and could also benefit other species.

## Conclusions

In conclusion, abdominal obesity, rather than overall obesity, was associated with heart disease in dogs. Increased relative waist circumference, especially a WIWDR greater than 3.6 or a WTLR greater than 1.2, may provide useful means of assessing cardiovascular disease risk status associated with the presence of an abdominal obesity in dogs. Because abdominal obesity puts dog at risk of serious medical problems including cardiovascular disease, veterinarians should raise awareness about the dangers of canine obesity and the long-term medical consequences.

## Abbreviations

AUC: Area under the curve; BCS: Body condition score; BW: Body weight; CT: Computed tomography; IAF: Intra-abdominal fat; MBMI: Modified body mass index; ROC: Receiver operating characteristic; SQF: Subcutaneous fat; TL: Truncal length; WHSDR: Waist-to-hock-to-stifle distance ratio; WIWDR: Waist-to-ilium wing distance ratio; WTLR: Waist-to-truncal length ratio.

## Competing interests

All authors declare that they have no competing interests.

## Authors’ contributions

NT research design, conduct research, data analysis, manuscript preparation; WT analysis of the computed tomographic data, manuscript revision; SK data analysis, manuscript revision; AS conduct research, manuscript revision. All authors read and approved the final manuscript.
